# Prophylactic hyperthermic intraperitoneal chemotherapy may benefit the long-term survival of patients after radical gastric cancer surgery

**DOI:** 10.1038/s41598-022-06417-y

**Published:** 2022-02-16

**Authors:** Xuhui Zhuang, Yuewen He, Wuhua Ma

**Affiliations:** Department of Anesthesiology, The First Affiliated Hospital of Guangzhou University of Chinese Medicine, 12 Jichang Road, Guangzhou, 510405 Guangdong People’s Republic of China

**Keywords:** Histocytochemistry, Immunochemistry, Diseases, Medical research, Oncology

## Abstract

Hyperthermic intraperitoneal chemotherapy (HIPEC) has been proven to improve the survival rate of gastric cancer and reduce peritoneal recurrence. We aimed to evaluate the effectiveness and safety of prophylactic HIPEC after radical gastric cancer surgery in this study. Researchers searched for studies published in PubMed, Embase, Web of science, Scopus, Cochrane, Clinical key databases and Microsoft Academic databases to identify studies that examine the impact of prophylactic HIPEC on the survival, recurrence and adverse events of patients undergoing radical gastric cancer surgery. RevMan 5.3 was used to analyze the results and risk of bias. The PROSERO registration number is CRD42021262016. This meta-analysis included 22 studies with a total of 2097 patients, 12 of which are RCTs. The results showed that the 1-, 3- and 5-year overall survival rate was significantly favorable to HIPEC (OR 5.10, 2.07, 1.96 respectively). Compared with the control group, the overall recurrence rate and peritoneal recurrence rate of the HIPEC group were significantly lower (OR 0.41, 0.24 respectively). Significantly favorable to the control group in terms of renal dysfunction and pulmonary dysfunction complications (OR 2.44, 6.03 respectively). Regarding the causes of death due to postoperative recurrence: liver recurrence, lymph node and local recurrence and peritoneal recurrence, the overall effect is not significantly different (OR 0.81, 1.19, 0.37 respectively). 1-, 3- and 5-year overall survival follow-up may be incremented by the prophylactic HIPEC, and which reduce the overall recurrence rate and peritoneal recurrence rate. HIPEC may have high-risk of pulmonary dysfunction and renal dysfunction complications. No difference has been found in the deaths due to recurrence after surgery.

## Introduction

Gastric cancer (GC) is not only one of the most common malignant tumors in the world, but also the malignant tumor with the second highest mortality rate among all kinds of tumors^1,2^. More than 70% of GC occur in developing countries, and more than 50% of cases occur in East Asia^3^. Liu et al.^4^ pointed out in a study published in 2020 that China’s annual morbidity and mortality of GC are twice the world average. At present, surgical resection is the only possible cure for gastric cancer^5^, however, the 5-year survival rate is still not satisfactory. Recurrence after GC treatment surgery is quite common, about 10–46% will have peritoneal recurrence after surgery^6,7^. Peritoneal dissemination is one of the main reasons for gastric cancer recurrence and metastasis in the abdominal cavity. And it will cause peritoneal cancer (PC), which is more complicated and harder to treat than GC.

Although some scholars have proposed in recent years that adjuvant chemotherapy and neoadjuvant chemotherapy can slightly improve the survival rate after radical gastric cancer surgery^7,8^, they have not shown to significantly reduce the distant metastasis rate. Despite the use of systemic chemotherapy and other methods, the survival rate of patients with advanced GC is still not ideal. It may be due to the existence of the "plasma-peritoneal barrier"^9,10^ that can isolate the abdominal cavity from the effect of intravenous chemotherapy, which leads to the poor response of PC and advanced GC to systemic chemotherapy. Some evidence in the peritoneal dialysis literature indicates that the peritoneal permeability of some hydrophilic anticancer drugs may be much lower than the plasma clearance rate. Pharmacokinetic calculations indicate that the concentration of this intraperitoneal ingested drug is expected to be much higher in the abdominal space than in the plasma^11^. At the same time, hyperthermia has been developed as an anti-cancer therapy. It is one of the most widely studied chemotherapy and radiotherapy sensitizers^12,13^, and it has been proven that it has a direct cytotoxic effect on tumor cells in the abdominal cavity in combination with certain anti-cancer chemotherapy. Therefore, a new combination therapy has been introduced in recent years, namely hyperthermic intraperitoneal perfusion chemotherapy (HIPEC), which is considered to be an effective method to control the peritoneal dissemination of GC patients after the radical GC surgery^2,14,15^. Since HIPEC has been proven effective for PC, peritoneal pseudomyxoma and other diseases, it has been included in the national treatment standards of some EU countries. But the safety and effectiveness of prophylactic HIPEC in patients with advanced gastric cancer and patients after radical gastric cancer surgery is still a hot topic of debates.

Can prophylactic HIPEC really improve the long-term survival rate of patients with radical GC? Effectively control peritoneal transmission? These are still the questions we want to explore. Therefore, this systematic review and meta-analysis will use the results of RCTs and high-quality NRCTs to comprehensively evaluate the effectiveness and safety of prophylactic HIPEC for patients after radical GC surgery in terms of short-term or long-term survival rate (1-, 3- and 5-years), recurrence rate, complications, and deaths due to recurrence after surgery.

## Methods

### Search strategy

**Figure 1:**
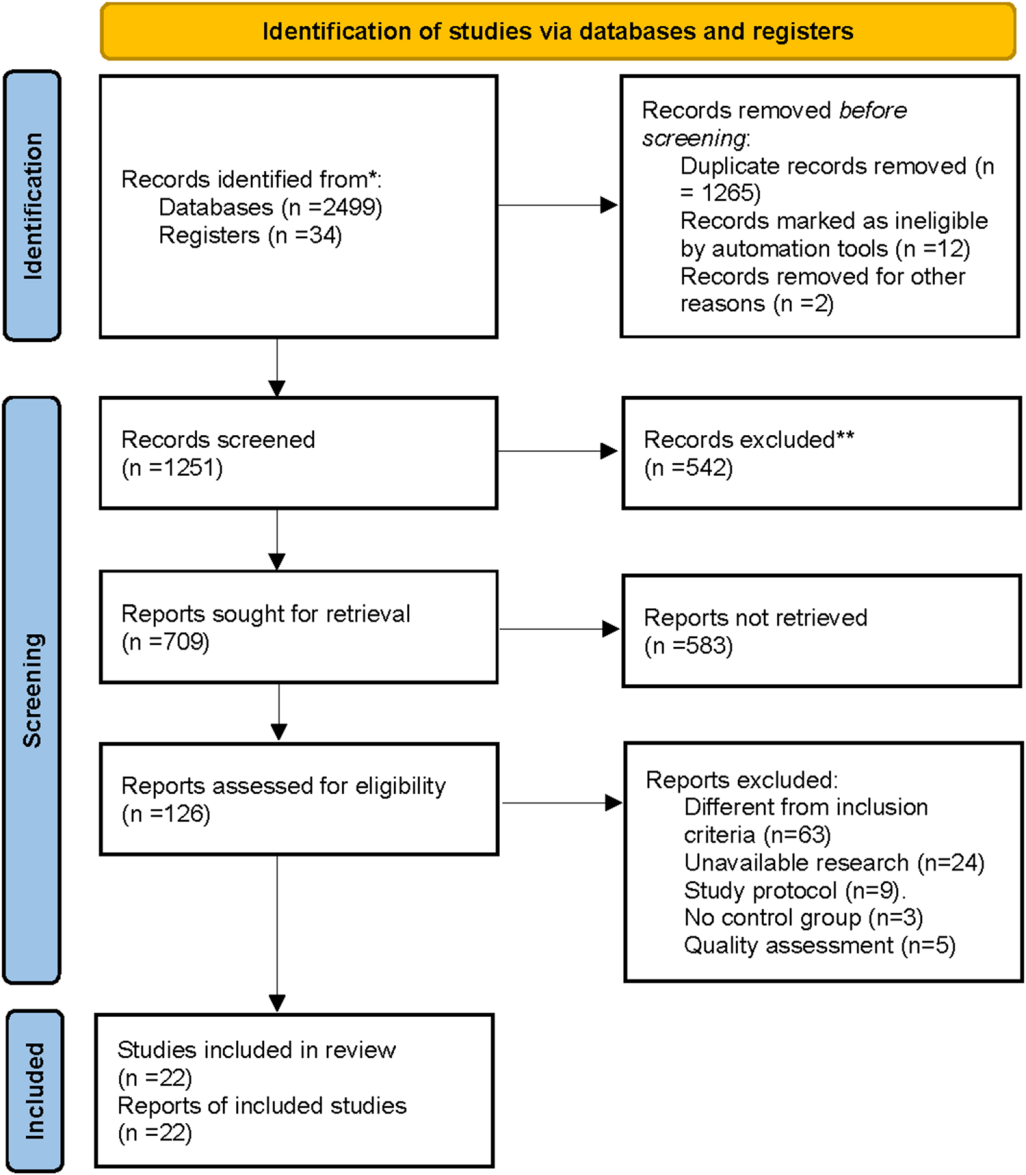
PRISMA Flow diagram of search strategy and included studies.

This review was conducted in accordance with the Preferred Reporting Items for Systematic Reviews and meta-analyses (PRISMA) guidelines, and we completed the PRISMA checklist according to the guidelines. Two investigators (X.H.Z, Y.W.H) searched for studies published in PubMed, Embase, Web of science, Scopus, Cochrane, and Clinicalkey databases from the inception to June 12, 2021. In addition, X.H.Z searched Microsoft Academic, and all search results are listed in PRISMA_2020_flow_diagram (Fig. 1). The researcher sets the search conditions as topic keywords and abstracts. There are no language restrictions throughout the search process. The search terms are: (HIPEC OR CHPP OR chemotherapeutic hyperthermic intraperitoneal perfusion OR intraperitoneal hyperthermic perfusion chemotherapy OR Peritoneal thermal perfusion OR Hyperthermic intraperitoneal perfusion OR IHPC OR CCCHP OR Coelom Continued Circulatory Hyperthermia Perfusion OR intraperitoneal chemohyperthermia) AND (gastric carcinoma OR gastric cancer OR stomach cancer OR Carcinoma of stomach OR radical gastrectomy for cancer OR Laparoscopic radical gastrectomy OR radical gastrectomy OR Radical operation of gastric carcinoma OR radical extremital partial gastrectomy OR radical operation for carcinoma of stomach OR radical correction for stomach cancer). We will change the search formula for different databases. In order to avoid omissions, we choose the search formula with the most search results.

### Study selection

The study selection process is carried out in EndNote X9 (Thomson Reuters, NY, USA). The entire retrieval process is divided into three parts. First, X.H.Z saves the respective search results of the two investigators to EndNote X9 and finds duplicates. After deleting all duplicate studies, X.H.Z will exclude studies marked as ineligible by automation tools or other reasons that cannot enter the second stage of screening. Subsequently, we screened out clinical studies and excluded Meta-analysis, Case reports, Reviews, Animal experiments, Letter, Laboratory studies, Guidelines, and conference abstract. The second stage is to screen the studies based on the topic, abstract and keywords. In this process, we use the Rating in EndNote X9 to rank the research. Two investigators marked the studies with “low relevance” as “one star”, “medium credibility” as “2–3 stars”, and “high credibility” as “4–5 star”. “The stars” determines the subsequent screening process. “One-star” research will be excluded at this stage, the “2–3 stars” needs to be re-evaluated by all investigators (X.H.Z Y.W.H W.H.M), and the “4–5 stars” can be included in the full text review. The third stage is the full-text review of the included studies. Two researchers excluded the studies of different from inclusion criteria, fail to obtain and protocol. We use Modified methodological index for non-randomized studies (MINORS) score^16,17^ to evaluate the quality of non-randomized control trails (NRCTs) and exclude studies with a total score of < 12. All disputes during the Study Selection process are resolved by the third investigator (W.H.M).

### Eligibility criteria

The purpose of this review is to evaluate the role of prophylactic HIPEC after radical resection of gastric cancer. Therefore, the inclusion criteria of the study are as follows: gastric cancer patients undergoing radical surgery, postoperative prophylactic HIPEC, blank control group or concurrent postoperative chemotherapy. And we excluded gastric cancer palliative surgical treatment, with peritoneal metastases, historical control, non-postoperative HIPEC, IPEC and non-chemotherapeutic intraperitoneal perfusion. Due to the small number of RCTs, we included some NRCTs and conducted quality assessments.

### Risk of bias assessment

**Figure 2:**
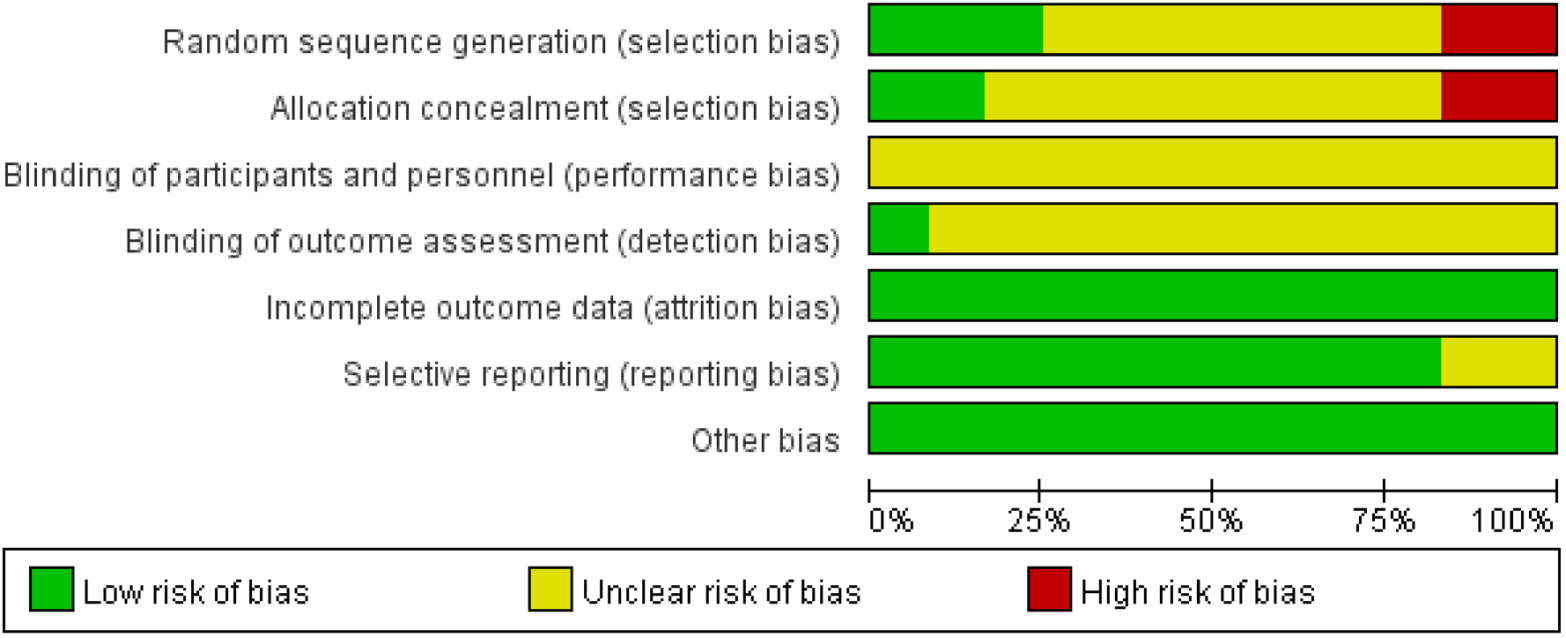
Risk of bias graph: review authors' judgements about each risk of bias item presented as percentages across all included studies.

**Figure 3:**
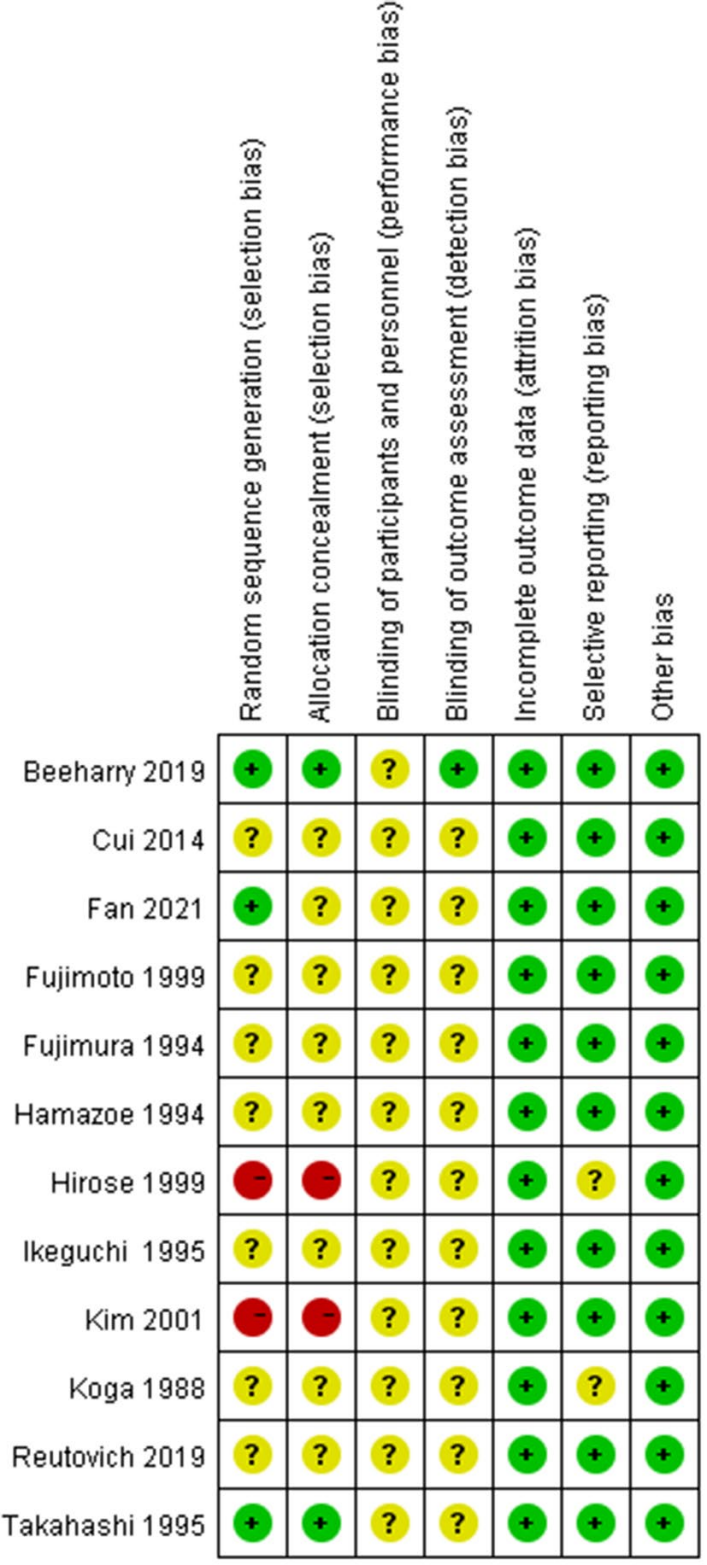
Risk of bias summary: review authors' judgements about each risk of bias item for each included study.

Two reviewers (X.H.Z, Y.W.H) used RevMan 5.3 (Review Manager. Version 5.3. Copenhagen: The Nordic Cochrane Centre, The Cochrane Collaboration, 2014.) to assess the risk of bias in RCTs. The authors' review of each risk of bias item's judgment is presented as a percentage of all included studies in Fig. 2. The authors' judgment of the risk of bias items for each of the included studies is shown in Fig. 3. The evaluation results are expressed as low risk, high risk and unclear. In case of dispute, W.H.M will determine the evaluation result.

### Data extraction

Two investigators (X.H.Z, Y.W.H) completed the data extraction independently, and W.H.M was responsible for handling different points of view. According to the Modified methodological index for non-randomized studies (MINORS) score, we will analyze the data included in the NRCT and complete the quality assessment Table 1. In addition, we extracted the characteristics of the studies and patients and summarized them in Tables 2, 3. The contents are as follows: Author, year of publication, Country, RCT/NRCT, study period, matched factors, ages, gender, and Cy + at the time of diagnosis in Table 2. And the Histologic type, T, N stage, etc. are shown in Table 3. The characteristics of the interventions will be summarized in Table 4. The patients’ prognosis and response to treatment are summarized in Table 5.

**Table 1:** Modified MINORS score of all eligible NRCT.

Author	Year		Consecutive patients		Prospective data collection	Reported endpoints	Unbiased outcome evaluation	Appropriate controls	Contemporary groups	Groups equivalent	Sample size	Score
Liu	2020		2		1	2	1	2	2	0	2	12
Xie	2020		2		1	2	1	2	2	1	2	13
Rosa	2021		1		1	2	2	2	2	1	1	12
Zhu	2020		1		1	2	2	2	2	2	1	13
Diniz	2020		2		2	2	2	2	2	1	1	14
Zhong	2020		2		1	2	2	2	2	2	2	15
Zhang	2020		1		1	2	1	1	2	1	2	11
Yonemura	1995		2		1	2	1	2	2	1	2	13
Hall	2004		1		1	1	1	2	2	1	2	11
Gao	2016		2		1	1	1	2	2	1	1	11
Kunisaki	2002		2		2	2	2	2	1	1	2	14
Kang	2013		2		1	2	2	2	2	2	1	14
Yarema	2014		2		1	2	2	2	2	1	1	13
Akiyama	2002		0		0	1	1	1	1	1	2	7
Kobayashi	1998		0		0	0	2	0	1	1	2	6

*MINORS* methodological index for non-randomised studies, *NRCT* Non-Randomized Controlled Trial.

Only studies with scores > 12 can be included in the meta-analysis.

**Table 2:** Summary of the characteristics of patients in 22 eligible studies.

Author Yr, country	RCT/NRCT	Matched factors	Study period	Group (n)	Average ages (Yr)	Gender, M/F	Cy + at the time of diagnosis
Liu 2020, China	NRCT	1246	January 2010 to April 2012	HIPEC (64)	69.4 (average)	68/60	NR
				Control (64)			NR
Cui 2014, China	RCT	12,346	January 2006 to January 2010	HIPEC (48)	53 (average)	22/26	NR
				Control (48)	56 (average)	21/27	NR
Fujimura 1994, Japan	RCT	123,456	March 1988 to March 1992	HIPEC (22)	60.2 (average)	12/10	NR
				Control (18)	62.9 (average)	10/8	NR
Fan 2021, China	RCT	1246	March 2015 to November 2016	HIPEC (33)	61 (average)	27/6	NR
				Control (17)	60 (average)	14/3	NR
Hamazoe 1994, Japan	RCT	12,346	January 1983 to October 1986	HIPEC (42)	56.5 ± 10.4 (mean ± SEM)	25/17	NR
				Control (40)	63.4 ± 9.6 (mean ± SEM)	31/9	NR
Xie 2020, China	NRCT	12,346	ND	HIPEC (51)	60.9 ± 7.1 (mean ± SD)	36/15	NR
				Control (62)	61.5 ± 8.6 (mean ± SD)	43/19	NR
Reutovich 2019, Belarus	RCT	12,346	2008 to 2016	HIPEC (68)	56 ± 8 (mean ± SD)	50/26	NR
				Control (55)	56 ± 9 (mean ± SD)	45/33	NR
Koga 1988, Japan	RCT	1246	July 1980 to February 1983	HIPEC (26)	NR	16/10	NR
				Control (21)	NR	17/4	NR
Rosa 2021, Italy	NRCT	1245	January 2006 to December 2015	HIPEC (23)	58 (mean)	11/12	NR
				Control (39)	68 (mean)	20/19	NR
Zhu 2020, China	NRCT	124	Jul 1, 2018 to Dec 31, 2019	HIPEC (22)	51 (median)	14/8	NR
				Control (21)	55 (median)	15/6	NR
Diniz 2020, Brazil	NRCT	12,456	2006 to 2017	HIPEC (28)	49.8 ± 10.8 (mean ± SD)	11/17	NR
				Control (56)	59.3 ± 11.3 (mean ± SD)	28/28	NR
Zhong 2020, China	NRCT	12,346	January 2016 to June 2017	HIPEC (61)	52.4 ± 10.7 (mean ± SD)	32/29	NR
				Control (68)	53.1 ± 10.5 (mean ± SD)	33/35	NR
Yonemura 1995, Japan	NRCT	12,345	1984 to 1992	HIPEC (79)	57.5 ± 11.7 (mean ± SD)	44/32	NR
				Control (81)	59.2 ± 13.6 (mean ± SD)	57/23	NR
Fujimoto 1999, Japan	RCT	1245	March 1987 to December 1996	HIPEC (71)	58.5 ± 8.1 (mean ± SD)	50/21	NR
				Control (70)	59.2 ± 9.1 (mean ± SD)	51/19	NR
Kim 2001, Korea	RCT	1246	1990 to 1995	HIPEC (52)	55.8 (mean)	68/35	NR
				Control (51)			NR
Hirose 1999, Japan	RCT	123,456	October 1988 to October 1995	HIPEC (15)	57, 41–65 (Median, IQR)	7/8	NR
				Control (40)	65, 56–73 (Median, IQR)	23/17	NR
Ikeguchi 1995, Japan	RCT	123,456	1980 to 1989	HIPEC (78)	62.6 ± 9.0 (mean ± SD)	43/35	NR
				Control (96)	61.2 ± 10.3 (mean ± SD)	64/32	NR
Takahashi 1995, Japan	RCT	12,346	January 1987 to December 1992	HIPEC (56)	55.7 (mean)	34/22	NR
				Control (57)	54.5 (mean)	34/23	NR
Beeharry 2019, China	RCT	12,346	December 2014 to June 2015	HIPEC (40)	59 ± 10 (mean ± SD)	23/17	0
				Control (40)	58 ± 10 (mean ± SD)	23/17	0
Kunisaki 2002, Japan	NRCT	123,456	April 1992 to March 1999	HIPEC (45)	53.0 ± 10.2 (mean ± SD)	32/13	0
				Control (79)	64.4 ± 10.5 (mean ± SD)	58/21	0
Kang 2013, Taiwan, ROC	NRCT	12,346	January 2002 to December 2010	HIPEC (29)	NR	NR	NR
				Control (83)	NR	NR	NR
Yarema 2014, Ukraine	NRCT	12,345	2008 to 2012	HIPEC (19)	NR	15/4	NR
				Control (19)	NR	13/6	NR

*HIPEC* hyperthermic intraperitoneal chemotherapy, *RCT* randomized control trial, *NRCT* Non-Randomized Controlled Trial, *ND* not declared, *M* male, *F* female, *IQR* interquartile range, *SD* standard deviation, *SEM* Standard Error of Mean, *Yr* year, Matching: 1, age; 2, gender; 3, histology; 4, stage; 5, lymphadenectomy; 6, type of gastrectomy.

**Table 3:** Summary of the characteristics of patients in 22 eligible studies.

Author Yr	Group	Histologic type			Stage			T stage	N stage
		Poorly or undifferentiated adenocarcinomas	Well or moderately differentiated adenocarcinomas	Mucinous adenocarcinoma or mucinous cell carcinoma	I/II	III	IV	1 ~ 3/4	0/1 ~ 3
Liu 2020	HIPEC & Control	60	50	18	NR	128	NR	NR	NR
Cui 2014	HIPEC	22	14	12	NR	48	NR	NR	NR
	Control	25	16	7	NR	48	NR	NR	NR
Fujimura 1994	HIPEC	NR	NR	NR	8	9	5	20/2	3/19
	Control	NR	NR	NR	5	5	8	15/3	2/15
Fan 2021	HIPEC	12	11	NR	NR	NR	NR	22/11	10/23
	Control	7	10	NR	NR	NR	NR	12/5	10/7
Hamazoe 1994	HIPEC	28	14	NR	13	16	5	NR	NR
	Control	29	11	NR	8	15	8	NR	NR
Xie 2020	HIPEC	NR	NR	NR	NR	NR	NR	6/56	8/54
	Control	NR	NR	NR	NR	NR	NR	7/44	6/45
Reutovich 2019	HIPEC	NR	NR	NR	0	68	0	T4: 76	23/53
	Control	NR	NR	NR	0	55	0	T4: 78	22/56
Koga 1988	HIPEC	NR	NR	NR	7	12	5	NR	8/18
	Control	NR	NR	NR	4	8	6	NR	6/15
Rosa 2021	HIPEC	NR	NR	NR	3	20	0	NR	1/22
	Control	NR	NR	NR	4	25	10	NR	6/33
Zhu 2020	HIPEC	NR	NR	2	4	18	NR	NR	NR
	Control	NR	NR	1	5	16	NR	NR	NR
Diniz 2020	HIPEC	NR	NR	NR	9	19	NR	10/18 (0 ~ 2/3 ~ 4)	7/21
	Control	NR	NR	NR	125	116	NR	124/117 (0 ~ 2/3 ~ 4)	137/104
Zhong 2020	HIPEC	NR	NR	NR	24	37	NR	37/24	35/26
	Control	NR	NR	NR	30	38	NR	38/30	43/25
Yonemura 1995	HIPEC	61	18	NR	15	32	32	NR	14/65
	Control	53	28	NR	29	17	35	NR	19/62
Fujimoto 1999	HIPEC	51	20	NR	NR	NR	NR	34/37	0/71
	Control	44	26	NR	NR	NR	NR	48/22	0/70
Kim 2001	HIPEC	22	30	NR	6	37	9	39/13	8/44
	Control	22	29	NR	19	28	4	47/4	9/42
Hirose 1999	HIPEC	14	1	NR	2	10	3	12/3	1/14
	Control	28	12	NR	12	21	7	31/9	9/31
Ikeguchi 1995	HIPEC	NR	NR	NR	NR	NR	NR	NR	18/60
	Control	NR	NR	NR	NR	NR	NR	NR	23/73
Takahashi 1995	HIPEC	41	9	6	6	26	24	NR	5/51
	Control	32	20	3	7	28	22	NR	2/55
Beeharry 2019	HIPEC	25 (Poor or moderately differentiated)		NR	NR	NR	NR	NR	NR
	Control	26 (Poor or moderately differentiated)		NR	NR	NR	NR	NR	NR
Kunisaki 2002	HIPEC	NR	NR	NR	11	17	17	45 (3 ~ 4)	11/34
	Control	NR	NR	NR	17	38	24	79 (3 ~ 4)	21/58
Kang 2013	HIPEC	96	25	NR	6	28	17	0/51	7/44
	Control	38	13	NR	8	73	40	0/121	12/109
Yarema 2014	HIPEC	17	2	NR	8	11	NR	0/19	NR
	Control	18	1	NR	11	8	NR	0/19	NR

*HIPEC* hyperthermic intraperitoneal chemotherapy, *NR* not recorded, *Yr* year.

**Table 4:** Summary of the treatments in the 22 eligible studies.

Author Yr, Country	Surgery	HIPEC group	Control group
Liu 2020, China	Radical gastrectomy	Surgery + HIPEC + systemic chemotherapy HIPEC: Oxaliplatin (100 mg/m^2^) and 0.9% sodium chloride solution (3000 mL) at 1.4 days; fluorouracil (0.75 g) and 0.9% sodium chloride solution (3000 mL) at 2.3 days; Dexamethasone (10 mg) and 2% lidocaine (10 mL) 1–4 days Time: postoperative 1 to 2 days (once a day, lasting 90 min each time), last for 4 days Temperature: 42–44 °C	Surgery + systemic chemotherapy (Paclitaxel 135 mg/m^2^(1 day), cisplatin 20 mg/m^2^ and tegafur 1.0 g (1–5 days), 4 weeks × 6 cycles) Time: 2 weeks postoperative
Cui 2014, China	Radical resection of the gastric cancer	Surgery + HIPEC + systemic chemotherapy HIPEC: Cisplatin (60 mg/m^2^) and 0.9% sodium chloride solution (3000 mL) at 1.4 days; fluorouracil (0.75 g) and 0.9% sodium chloride solution (3000 mL) at 2.3 days; Dexamethasone (10 mg) and 2% lidocaine (10 mL) 1–4 days Time: postoperative 1 to 2 days (once a day, lasting 90 min each time), last for 4 days Temperature: 41–43 °C	Surgery + ECF (50 mg/m^2^ epirubicin and 60 mg/m^2^ cisplatin administered via an intravenous drip on day 1 and 600 mg/m^2^ fluorouracil administered via an intravenous drip between day 1 and 3)
Fujimura 1994, Japan	Curative resection	Surgery + HIPEC HIPEC: MMC (30 mg) and cisplatinum (300 mg) in 10 L saline solution Time: 60 min Temperature: 41–42 °C	Surgery
Fan 2021, China	Radical gastrectomy	Surgery + HIPEC + chemotherapy with SOX regime HIPEC: Cisplatin (50 mg/L) and 0.9% sodium chloride solution Time: 30 min Temperature: 42.5–43 °C	Surgery + chemotherapy with SOX regime (S-1, 40–60 mg (40 mg when BSA < 1.25 m^2^, 60 mg when BSA > 1.5 m^2^), twice per day, Day 1–14; Oxaliplatin (130 mg/m^2^) was given intravenously at the first day of each cycle)
Hamazoe 1994, Japan	Radical gastrectomy	Surgery + HIPEC HIPEC: MMC (10 μg/mL) in 0.9% saline solution Time: 50–60 min Temperature: 44–45 °C	Surgery
Xie 2020, China	Laparoscopic-assisted radical gastrectomy	Surgery + HIPEC + systemic chemotherapy (SELOX or SOX) HIPEC: Cisplatin (50 mg/L) and 0.9% sodium chloride solution Time: 60 min Temperature: 42–43 °C	Surgery + XELOX or SOX chemotherapy at 4–6 weeks after surgery and received a total of 6–8 cycles every 3 weeks. (Regimen: Oxaliplatin 130 mg/m^2^ ivgtt d1 + xeloda 1500 mg/m^2^ BID PO d1–15))
Reutovich 2019, Belarus	Total or partial (distal subtotal resection) gastrectomy with free margins (R0 resection) and D2 lymph node dissection	Surgery + HIPEC HIPEC: Ringer's solution (5–6 L), cisplatin (50 mg/m^2^) and doxorubicin (50 mg/m^2^) Temperature: 42 °C	Surgery
Koga 1988, Japan	Curative surgery	Surgery + HIPEC HIPEC: MMC (8–10 mg/L) in 2000 mL saline solution Time: 50–60 min Temperature: 44–45 °C	Surgery
Rosa 2021, Italy	Gastrectomy	Surgery + HIPEC HIPEC: Cisplatin (75 mg/m^2^), MMC (15 mg/m^2^), and 0.9% sodium chloride solution (2 L/m^2^) Time: 90 min Temperature: 41–42 °C	Surgery
Zhu 2020, China	Gastrectomy	Surgery + HIPEC + chemotherapy HIPEC: Cisplatin (75 mg/m^2^) and 2000 mL 0.9% sodium chloride solution Time: 60 min Temperature: 41.5–42.5 °C	Surgery + chemotherapy intravenous 5-fluorouracil (500 mg/m^2^) and LV (200 mg/m^2^) on days 1 to 5, and intravenous cisplatin (25 mg/m^2^) on days 1 to 3
Diniz 2020, Brazil	Curative resection	Perioperative chemotherapy + surgery + HIPEC HIPEC: MMC (38 mg/m^2^) in saline solution Time: 90 min Temperature: 41–42 °C	Perioperative chemotherapy + surgery (a) Platinum‐based doublets (Carboplatin + Paclitaxel, Carboplatin + 5‐FU, CDDP + 5‐FU, FOLFOX, XELOX, FLOX) (b) Epirubicin‐based triplets (ECF, ECX, EOX) (c) Taxane‐based triplets (DCF, DCX)
Zhong 2020, China	Laparoscopic-assisted radical gastrectomy	Surgery + HIPEC HIPEC: Lobaplatin (50 mg/m^2^) and 3000 mL 5% intravenous glucose solution Time: 60 min Temperature: 43 °C	Surgery + chemotherapy 400 mg UFT [a combination of 1-(2-tetrahydrofuryl)-5-fluorouracil and uracil in a molar ratio of 1:4] per day orally on consecutive days for the first 2 to 3 postoperative weeks
Yonemura 1995, Japan	Gastrectomy	Surgery + HIPEC HIPEC: Cisplatin (300 mg), MMC (30 mg), and 0.9% sodium chloride solution (8 L) Time: 60 min Temperature: 41.5–43.5 °C	Surgery
Fujimoto 1999, Japan	Gastrectomy	Surgery + HIPEC HIPEC: MMC (10 mg/mL), and 0.9% sodium chloride solution (3–4 L) Time: 120 min Temperature: 44.5–45 °C	Surgery
Kim 2001, Korea	Subtotal or total gastrectomy	Surgery + HIPEC HIPEC: MMC (40 mg), and dialysis solution (4000 cc) Time: 120 min Temperature: 44.5–45.7 °C	Surgery + chemotherapy 5-fluorouracil (FU) or 5-FU in combination with MMC at least six cycles
Hirose 1999, Japan	Gastrectomy	Surgery + HIPEC HIPEC: cisplatin (100 mg), MMC (20 mg) and etoposide (100 mg) Temperature: 41–44.5 °C	Surgery + chemotherapy Two to 3 weeks after the operation, MMC (6 mg/m^2^) and 5-fluorouracil (5FU, 375 mg/m^2^) were intravenously administered once a week, and this MMC-5FU therapy was repeated for 3 consecutive weeks before the patient’s discharge from the hospital
Ikeguchi 1995, Japan	Curative resection	Surgery + HIPEC HIPEC: MMC (80–100 mg/m^2^), 8–10 L Time: 50–60 min Temperature: 44–45 °C	Surgery + chemotherapy intravenous injection of MMC 20 mg on day 0 and MMC 10 mg on days 7 and 14, and took 1-(2tetrahydrofuryl)-5-fluorouracil/uracil (1:4) 600 mg/day orally from day 14 for at least 6 months
Takahashi 1995, Japan	Gastrectomy	Surgery + HIPEC HIPEC: MMC (50 mg), Activated carbon (375 mg), Saline (100 mL)	Surgery
Beeharry 2019, China	Standardized radical gastrectomy with D2 lymphadenectomy	Surgery + HIPEC HIPEC: cisplatin (50 mg/L) Time: 60 min Temperature: 41–43 °C	Surgery + XELOX 6 regimens of standard dosage of the XELOX regimen starting within 1 month after surgery (Regimen: Oxaliplacin 130 mg/m^2^ ivgtt d1 + Xeloda 1500 mg/m^2^bid po d1–15, Q3W)
Kunisaki 2002, Japan	Gastrectomy	Surgery + HIPEC HIPEC: Cisplatin (150 mg), MMC (15 mg), and etoposide (150 mg) in 5 to 6 L physiologic saline Time: 40 min Temperature: 42–43 °C	Surgery
Kang 2013, Taiwan, ROC	Gastrectomy	Surgery + HIPEC HIPEC: cisplatin (30 mg/L), MMC (10 mg/L), and etoposide (20 mg/L) in 3–4 L of lactated Ringer solution Time: 60 min Temperature: 41–43 °C	Surgery
Yarema 2014, Ukraine	Gastrectomy	Surgery + HIPEC HIPEC: MMC (12.5 mg/m^2^), cisplatin (75 mg/m^2^) Time: 90 min Temperature: 41–43.6 °C	Surgery

*HIPEC* hyperthermic intraperitoneal chemotherapy, *Yr* year, *MMC* mitomycin C.

**Table 5:** The patients’ prognosis and response to treatment in the 22 eligible studies.

Author Yr	Group	Survival rate			Recurrence			Complication		
		1-year, %	3-year, %	5-year, %	Recurrence rate, %	Recurrence rate: peritoneal, %	Myelosuppression, n	Leakage, n	Liver dysfunction, n	Renal dysfunction, n
Liu 2020	HIPEC (64)	96.88	70.31	28.13	7.81	NR	NR	NR	NR	NR
	Control (64)	79.69	34.38	9.38	25.00	NR	NR	NR	NR	NR
Cui 2014	HIPEC (48)	85.41	58.33	NR	16.67	NR	27	NR	NR	NR
	Control (48)	79.16	35.41	NR	33.33	NR	26	NR	NR	NR
Fujimura 1994	HIPEC (22)	95.45	90.91	NR	NR	25	4	1	2	NR
	Control (18)	44.44	22.22	NR	NR	25	NR	NR	NR	NR
Fan 2021	HIPEC (33)	NR	87.90	NR	NR	NR	2	4	23	NR
	Control (17)	NR	100.00	NR	NR	NR	2	1	13	NR
Hamazoe 1994	HIPEC (42)	NR	NR	64.29	NR	NR	NR	2	NR	NR
	Control (40)	NR	NR	52.50	NR	NR	NR	3	NR	NR
Xie 2020	HIPEC (51)	96.08	68.63	NR	21.57	3.92	7	0	NR	3
	Control (62)	95.16	66.13	NR	46.77	17.74	7	0	NR	2
Reutovich 2019	HIPEC (68)	NR	47.37	NR	52.9	12.8	Surgery-related complications: 9 Nonsurgical complications: 11			
	Control (55)	NR	26.92	NR	76.4	27.6	Surgery-related complications: 5 Nonsurgical complications: 7			
Koga 1988	HIPEC (26)	NR	73.08	NR	NR	NR	NR	1	NR	NR
	Control (21)	NR	52.38	NR	NR	NR	NR	2	NR	NR
Rosa 2021	HIPEC (23)	NR	NR	34.78	NR	21.74	NR	1	NR	NR
	Control (39)	NR	NR	10.26	NR	66.67	NR	4	NR	NR
Zhu 2020	HIPEC (22)	NR	NR	NR	63.64	4.55	NR	NR	12	8
	Control (21)	NR	NR	NR	90.48	33.33	NR	NR	7	4
Diniz 2020	HIPEC (28)	NR	NR	NR	46.43	28.57	NR	NR	NR	NR
	Control (56)	NR	NR	NR	21.99	9.54	NR	NR	NR	NR
Zhong 2020	HIPEC (61)	NR	89.4	NR	NR	4.92	NR	3	0	0
	Control (68)	NR	84.3	NR	NR	17.65	NR	3	1	0
Yonemura 1995	HIPEC (79)	NR	NR	48.15	NR	NR	NR	NR	NR	NR
	Control (81)	NR	NR	35.44	NR	NR	NR	NR	NR	NR
Fujimoto 1999	HIPEC (71)	NR	NR	NR	NR	1.41	0	2	NR	NR
	Control (70)	NR	NR	NR	NR	22.86	0	2	NR	NR
Kim 2001	HIPEC (52)	NR	NR	32.69	69.23	13.46	2	1	NR	1
	Control (51)	NR	NR	27.45	68.63	29.41	0	2	NR	0
Hirose 1999	HIPEC (15)	NR	46.67	40.00	53.33	26.67	6 (overall)	3	8 (overall)	3
	Control (40)	NR	30.00	17.50	67.50	45.00	8(overall)	6	13 (overall)	5
Ikeguchi 1995	HIPEC (78)	NR	NR	50.64	NR	34.62	NR	NR	NR	NR
	Control (96)	NR	NR	45.74	NR	39.58	NR	NR	NR	NR
Takahashi 1995	HIPEC (56)	NR	37.50	NR	NR	NR	5	3	NR	NR
	Control (57)	NR	19.30	NR	NR	NR	1	2	NR	NR
Beeharry 2019	HIPEC (40)	NR	NR	NR	NR	2.50	1	0	0	1
	Control (40)	NR	NR	NR	NR	22.50	2	1	2	1
Kunisaki 2002	HIPEC (45)	NR	57.78	48.89	NR	50.00	NR	1	1	3
	Control (79)	NR	56.96	54.43	NR	67.74	NR	2	1	0
Kang 2013	HIPEC (29)	NR	NR	44.83	NR	NR	NR	NR	NR	NR
	Control (83)	NR	NR	10.84	NR	NR	NR	NR	NR	NR
Yarema 2014	HIPEC (19)	100.00	NR	NR	NR	10.53	NR	NR	NR	NR
	Control (19)	52.63	NR	NR	NR	73.68	NR	NR	NR	NR

*HIPEC* hyperthermic intraperitoneal chemotherapy, *NR* not record, *Yr* year.

### Outcomes

The primary outcome of this review is the overall survival at 3 years follow-up. The secondary outcomes are the overall survival at 1- and 5-years follow-up; recurrence rate: overall and peritoneal; complication: myelosuppression, leakage, intestinal obstruction, liver dysfunction; deaths due to recurrence after surgery: liver, lymph node and local and peritoneal recurrence.

### Statistical analysis

All the data that needs to be analyzed are dichotomous data, and we choose to report odds ratio (OR). RevMan 5.3 also reported the heterogeneity of the data while producing the forest plot. For heterogeneity test P < 0.05 or I_2_ > 50%, we choose random effects model. When the heterogeneity test P > 0.05 or I_2_ < 50%, the fixed effects model is often selected. Subgroup analysis is based on the overall heterogeneity inspection results. The fixed effects model is used when the results of heterogeneity between subgroups are consistent, and the random effects model is used when the results of heterogeneity are inconsistent. If the heterogeneity test result I^2^ > 80%, we need to perform a sensitivity analysis on the data to exclude studies with significant heterogeneity.

## Results

### Literature search findings

Two researchers (X.H.Z Y.W.H) searched PubMed, Embase, Web of science, Scopus, Cochrane, Clinicalkey, and Microsoft Academic databases, and a total of 2533 studies were obtained. X.H.Z used EndNote X9 to remove 1268 duplicate studies. We excluded 12 records marked as ineligible by automation tools and 2 studies due to incomplete information. And two investigators independently reviewed the initially included studies and excluded 542 non-clinical studies (Review: 349; Meta-analysis: 26; Case report: 42; Letter: 28; Animal experiments: 26; Laboratory studies: 26; Guidelines or Conference Abstract: 45). Based on the number of "stars" marked in EndNote X9, we screened clinical studies in the second stage, and 126 studies can be reviewed in full text. After excluding 99 studies, 27 included articles were identified. We evaluated the quality of NRCT among them, three studies with a score of < 12 were excluded^18–22^. This review finally included 22 articles. The literature search findings are represented in PRISMA_2020_flow_diagram (Fig. 1).

### Study and patient characteristics

The characteristics of the included studies are summarized in Tables 2, 3. A total of 22 studies (RCT:12 NRCT:10) with 2097 patients were included in this review. Among them, 9 studies were published after 2015. Most of the included studies are from Asia, including 8 from mainland China^23–29^ and Taiwan ROC^30^, 10 from Japan^31–39^ and Korea^40^. The other three studies are from Belarus^41^, Italy^42^, Ukraine^43^, and Brazil^44^. Matched factors mainly include the following: age, gender, histology, stage, lymphadenectomy, type of gastrectomy. If the above content is reported in the research, the investigators will use the corresponding number of the representative to express it. The included studies all reported the patient’s age, gender, and stage of gastric cancer. For the staging of gastric cancer, 8 studies^25,27,30,34,35,37,40,41^ reported the TMN staging situation, and 15 studies^23,24,26,28,29,31–33,36,38–40,42–44^ listed the number of patients in each stage. The studies of Liu et al.^26^, Cui et al.^28^, and Reutovich et al.^41^ included only stage III patients.

### Intervention characteristics

Two investigators summarized the intervention characteristics of the included studies in Table 3. In the included studies, the choice of chemotherapeutics for HIPEC was mainly MMC or Cisplatin or a combination of the two. The HIPEC protocol chosen by cui and his colleagues^28^ is as follows: Cisplatin (60 mg/m^2^) and 0.9% sodium chloride solution (3000 mL) at 1.4 days; fluorouracil (0.75 g) and 0.9% sodium chloride solution (3000 mL) at 2.3 days; Dexamethasone (10 mg) and 2% lidocaine (10 mL) 1–4 days; temperature: 41–43 °C. Xie et al.^25^ used Cisplatin (50 mg/L) and 0.9% sodium chloride solution for 60 min, temperature: 42–43 °C. Reutovich et al.^41^ chose Ringer's solution (5–6 L), cisplatin (50 mg/m^2^) and doxorubicin (50 mg/m^2^), Temperature: 42 °C. Zhu et al.'s^23^ solution is: Cisplatin (75 mg/m^2^) and 2000 mL 0.9% sodium chloride solution for 60 min, temperature: 41.5–42.5 °C. Beeharry et al.^29^ and his colleagues used cisplatin (50 mg/L) for 60 min, temperature: 41–43 °C. The researchers used MMC chemotherapy in 8 studies. The method of Hamazoe et al.^33^ is: MMC (10 μg/mL) in 0.9% saline solution for 50–60 min, temperature: 44–45 °C. Koga et al.^32^ chose MMC (8–10 mg/L) in 2000 mL saline solution for 50–60 min, temperature: 44–45 °C. Diniz et al.^44^ used MMC (38 mg/m^2^) in saline solution for Time: 90 min, temperature: 41–42 °C. Fujimoto and his colleagues^35^ chose MMC (10 mg/mL), and 0.9% sodium chloride solution (3–4 L) for 120 min, temperature: 44.5–45 °C. Kim et al.^40^ chose MMC (40 mg) and dialysis solution (4000 cc) for 60 min, temperature: 44.5–45.7 °C in RCT. Ikeguchi et al.^37^ chose MMC (80–100 mg/m^2^) for 50–60 min, temperature: 44–45 °C. Takahashi et al.^38^ method is: MMC (50 mg), Activated carbon (375 mg), Saline (100 mL). In addition, 7 studies chose the HIPEC scheme of MMC + cisplatin. Fujimura et al.^34^ used MMC (30 mg) and cisplatinum (300 mg) in 10 L saline solution for 60 min, temperature: 41–42 °C. Rosa et al.^42^ used Cisplatin (75 mg/m^2^), MMC (15 mg/m^2^), and 0.9% sodium chloride solution (2 L/m^2^) for 90 min, temperature: 41–42 °C. Yonemura et al.^31^ and his colleagues used Cisplatin (300 mg), MMC (30 mg), and 0.9% sodium chloride solution (8 L) for 60 min, temperature: 41.5–43.5 °C. Hirose et al.^36^ reported cisplatin (100 mg), MMC (20 mg) and etoposide (100 mg), temperature: 41–44.5 °C. Kunisaki et al.^39^ chose cisplatin (150 mg), MMC (15 mg), and etoposide (150 mg) in 5 to 6 L physiologic saline for 40 min, temperature: 42–43 °C. Kang et al.^30^ chose cisplatin (30 mg/L), MMC (10 mg/L), and etoposide (20 mg/L) in 3–4 L of lactated Ringer solution for 60 min, temperature: 41–43 °C. Yarema et al.^43^ used MMC (12.5 mg/m^2^), cisplatin (75 mg/m^2^) for 90 min, temperature: 41–43.6 °C. In addition, the study by Liu et al.^26^ used Oxaliplatin (100 mg/m^2^) and 0.9% sodium chloride solution (3000 mL) at 1.4 days; fluorouracil (0.75 g) and 0.9% sodium chloride solution (3000 mL) at 2.3 days; Dexamethasone (10 mg) and 2% lidocaine (10 mL) 1–4 days, temperature: 42–44 °C. Zhong et al.^24^ used Lobaplatin (50 mg/m^2^) and 3000 mL 5% intravenous glucose solution for 60 min, Temperature: 43 °C.

### Risk of bias assessment and study quality

**Figure 4:**
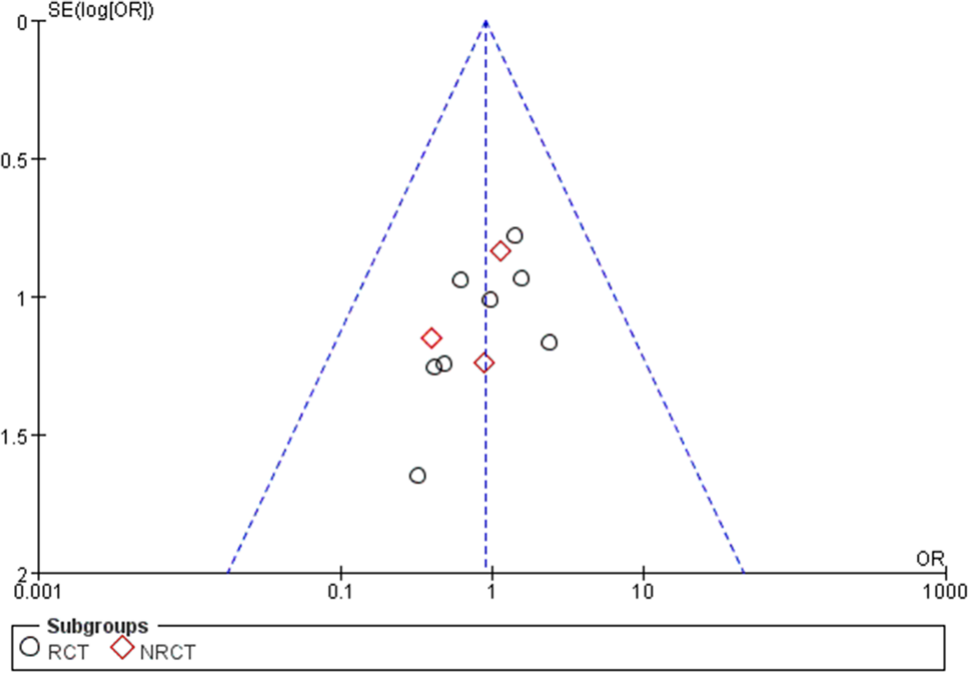
Funnel plot of comparison the complications.

Two investigators used RevMan 5.3 to assess the risk of bias for 12 RCTs. The evaluation result is shown in Figs. 2 and 3. Among all the included RCTs, 3 studies^27,29,38^ reported the method of random sequence generation, Beeharry et al.^29^ and Takahashi et al.^38^ reported on the concealment of random sequences. Hirose et al.^36^ and Kim et al.^40^ pointed out in the study that random sampling cannot be achieved due to the particularity of interventions. Although we evaluate this as high risk, this cannot be the basis for excluding these two studies. Only Beeharry et al. reported blinding the researchers responsible for data statistics, and none of the other studies mentioned blinding. In addition, the simple size is small in two studies^32,36^, and there may be a risk of reporting bias. In addition, the Funnel plot is used to assess the publication bias of the study (Fig. 4).

### Meta-analysis and synthesis

#### Overall 1-year survival (Fig. 5A)

**Figure 5:**
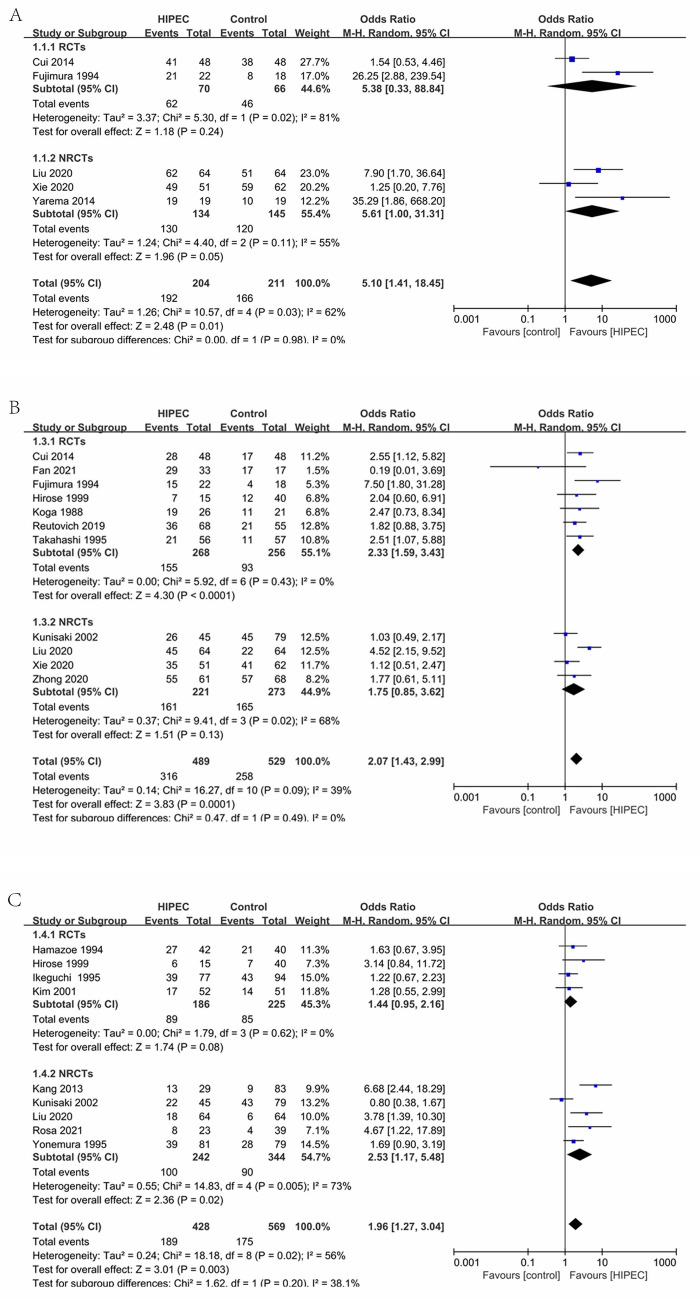
Overall 1-year survival (**A**), Overall 3-year survival (**B**), Overall 5-year survival (**C**).

Four studies (2RCTs, 3NRCTs), 415 patients were reported overall 1-year survival^25,26,28,34,43^. Analyzing under the random effects model, the overall heterogeneity (I_2_ = 62%) is acceptable. The Overall 1-year survival rate was significantly favorable to the HIPEC (OR 5.10, 95% CI 1.41–18.45).

#### Overall 3-year survival (Fig. 5B)

Ten studies (7RCTs, 4NRCTs), 1018 patients were reported overall 3-year survival^24–28,32,34,36,38,39,41^. Analyzing under the random effects model, the overall heterogeneity (I_2_ = 39%) is acceptable. The Overall 3-year survival rate was significantly favorable to the HIPEC (OR 2.07, 95% CI 1.43–2.99).

#### Overall 5-year survival (Fig. 5C)

Seven studies (4RCTs, 5NRCTs), 997 patients were reported overall 5-year survival^26,30,31,33,36,37,39,40,42^. Analyzing under the random effects model, the overall heterogeneity (I_2_ = 56%) is acceptable. The Overall 5-year survival rate was significantly favorable to the HIPEC (OR 1.96, 95% CI 1.27–3.04).

#### Overall 3-year survival in different HIPEC ways (Fig. 6)

**Figure 6:**
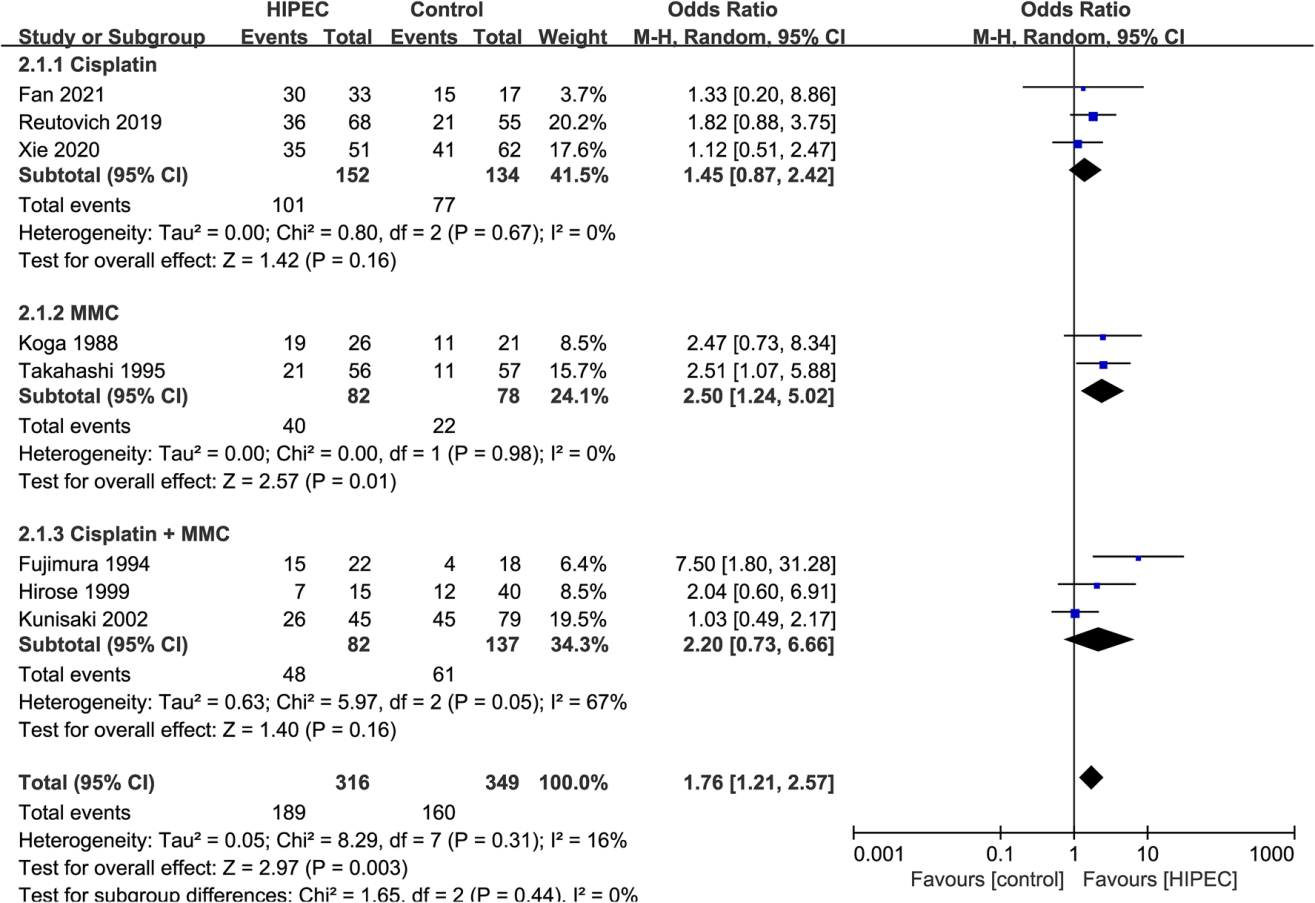
Overall 3-year survival in different HIPEC ways.

Eight studies (3Cisplatin, 2MMC, 3Cisplatin + MMC), 665 patients were reported overall 3-year survival in different HIPEC ways. Analyzing under the random effects model, the overall heterogeneity (I_2_ = 16%) is acceptable. The overall 3-year survival in different HIPEC ways was significantly favorable to the HIPEC (OR 1.76, 95% CI 1.21–2.57).

#### Overall recurrence rate (Fig. 7A)

**Figure 7:**
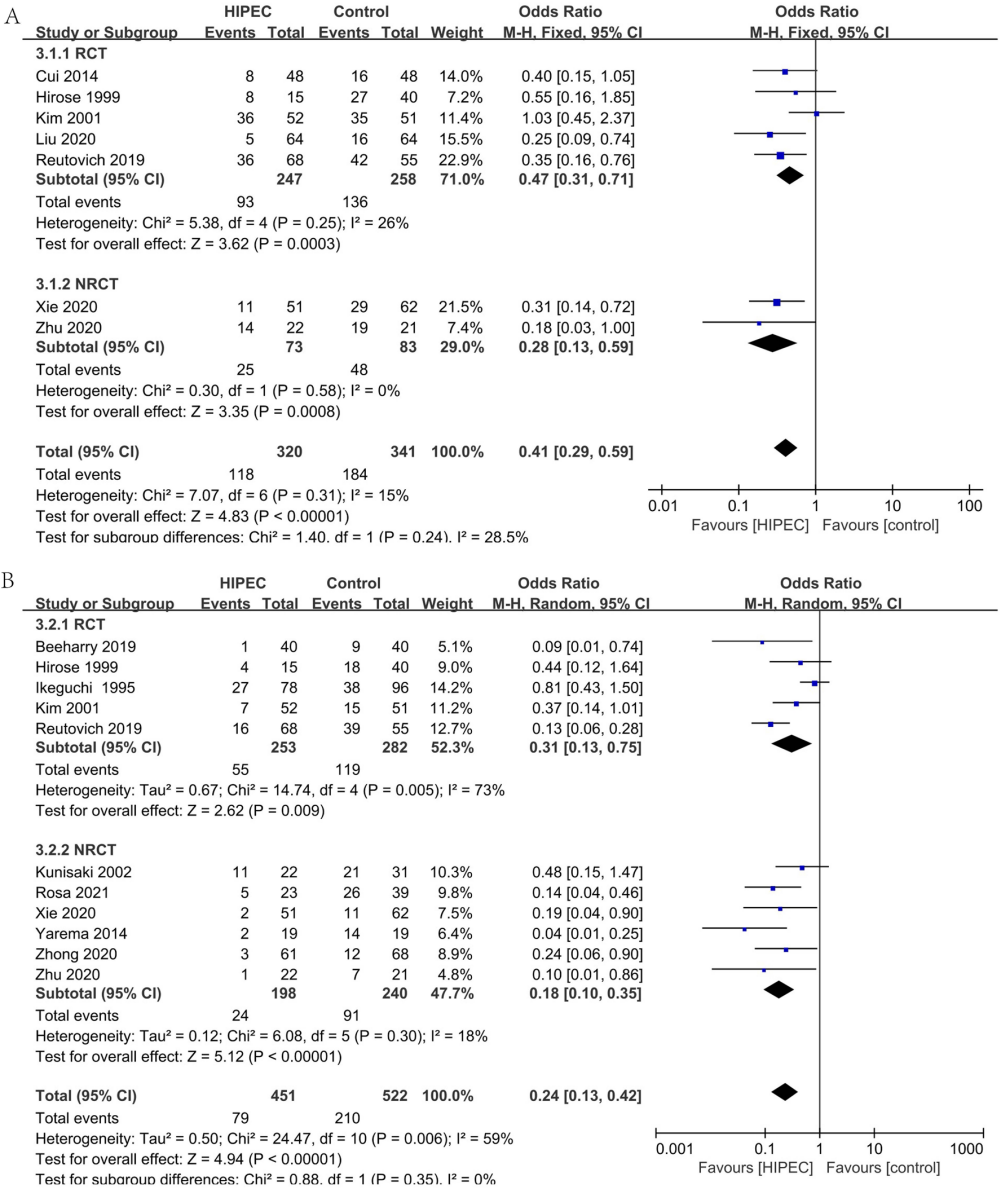
Overall recurrence rate (**A**), Peritoneal recurrence rate (**B**).

Eight studies, 930 patients were reported overall recurrence rate^23,25,26,28,36,40,41,44^. Using random effects model analysis, the heterogeneity is significant. Therefore, we conducted a sensitivity analysis and finally excluded studies^44^ that caused significant heterogeneity. Seven studies (5RCTs, 2NRCTs) with 661 patients were evaluated. Using fixed effects model analysis, the heterogeneity is no longer significant (I_2_ = 15%). The overall recurrence rate was significantly favorable to the HIPEC (OR 0.41, 95% CI 0.29–0.59).

#### Peritoneal recurrence rate (Fig. 7B)

Twelve studies, 1242 patients were reported peritoneal recurrence rate^23–25,29,36,37,39–43^. Using random effects model analysis, the heterogeneity is significant. The previous sensitivity analysis has excluded study with significant heterogeneity^44^. Eleven studies (5RCTs, 6NRCTs) with 973 patients were evaluated. Using random effects model analysis, the heterogeneity is no longer significant (I_2_ = 59%). The peritoneal recurrence rate was significantly favorable to the HIPEC (OR 0.24, 95% CI 0.13–0.42).

#### Complication: myelosuppression (Fig. 8A)

**Figure 8:**
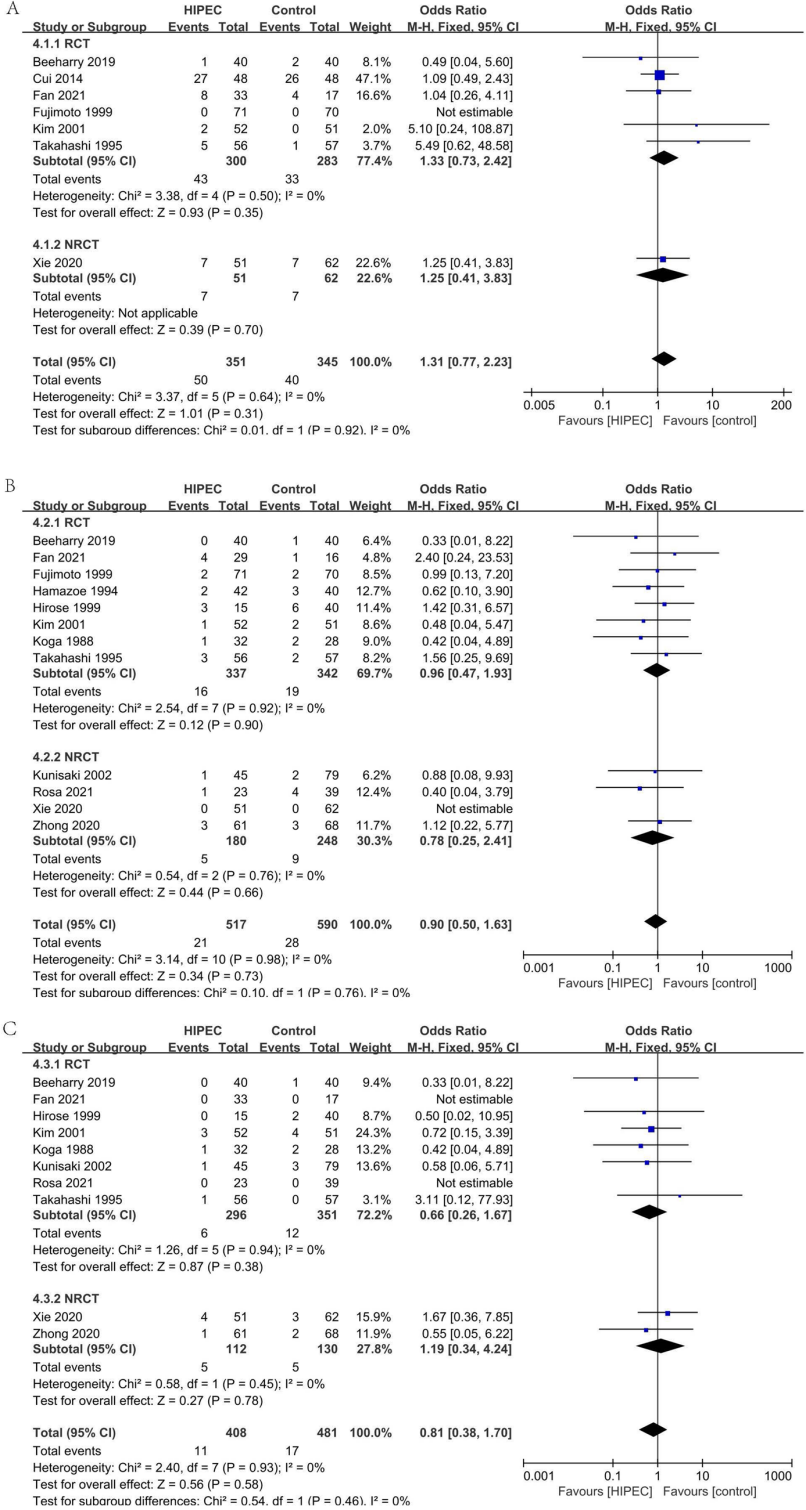
Complication: myelosuppression (**A**), Complication: leakage (**B**), Complication: intestinal obstruction (**C**).

In seven studies (6RCTs, 1NRCT), 696 patients reported the incidence of postoperative myelosuppression^25,27–29,35,38,40^. Analyzing under the fixed effects model, the overall heterogeneity (I_2_ = 0%) is not significant. The overall effect is not significantly different (OR 1.31, 95% CI 0.77–2.23).

#### Complication: leakage (Fig. 8B)

In twelve studies (8RCTs, 4NRCTs), 1107 patients reported the incidence of postoperative leakage^24,25,27,29,32,33,35,36,38–40,42^. Analyzing under the fixed effects model, the overall heterogeneity (I_2_ = 0%) is not significant. The overall effect is not significantly different (OR 0.90, 95% CI 0.50–1.63).

#### Complication: intestinal obstruction (Fig. 8C)

In ten studies (8RCTs, 2NRCTs), 889 patients reported the incidence of postoperative intestinal obstruction^24,25,27,29,32,36,38–40,42^. Analyzing under the fixed effects model, the overall heterogeneity (I_2_ = 0%) is not significant. The overall effect is not significantly different (OR 0.81, 95% CI 0.38–1.70).

#### Complication: liver dysfunction (Fig. 9A)

**Figure 9:**
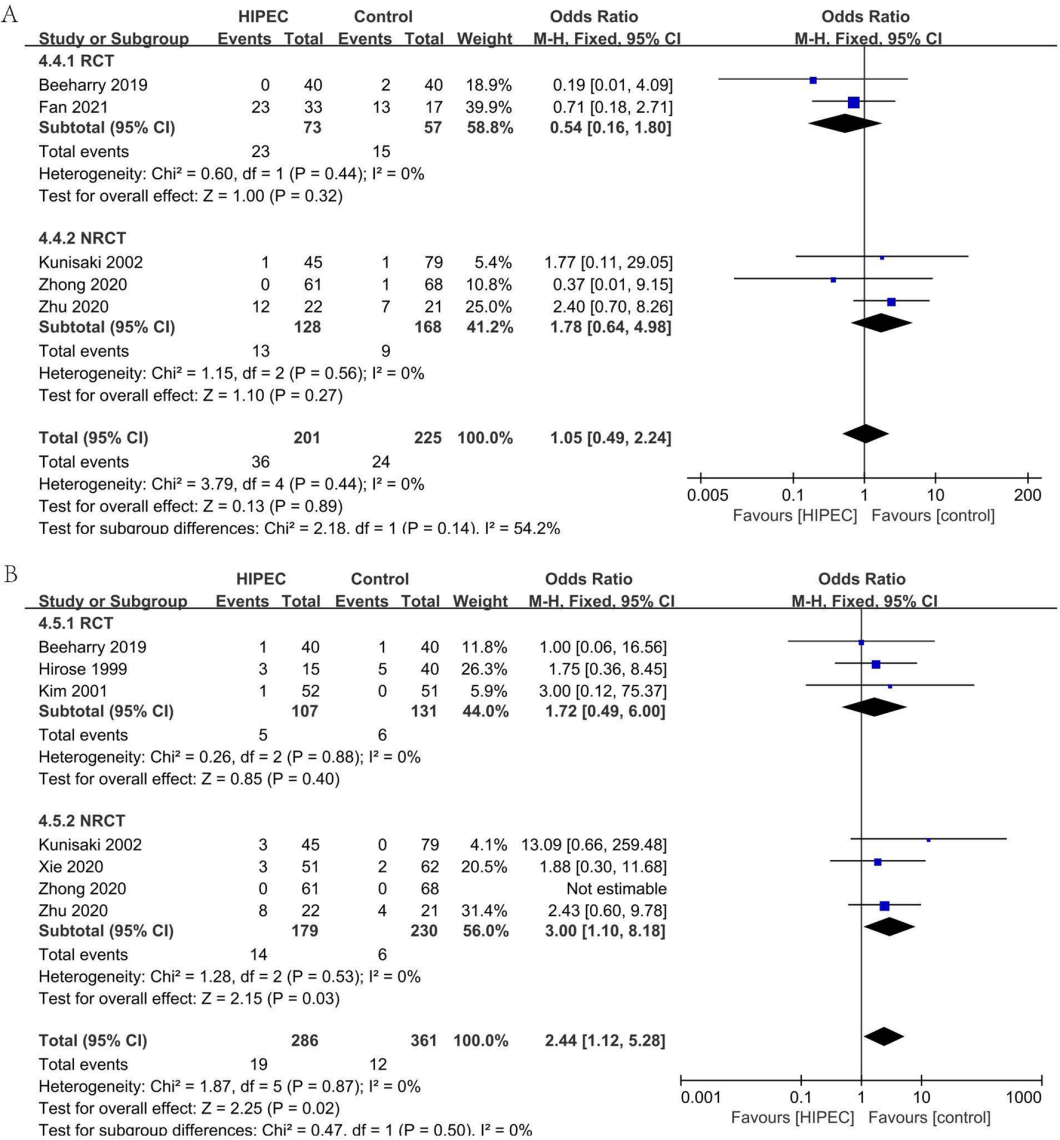
Complication: liver dysfunction (**A**), Complication: renal dysfunction (**B**).

In five studies (2RCTs, 3NRCTs), 426 patients reported the incidence of postoperative liver dysfunction^23,24,27,29,39^. Analyzing under the fixed effects model, the overall heterogeneity (I_2_ = 0%) is not significant. The overall effect is not significantly different (OR 1.05, 95% CI 0.49–2.24).

#### Complication: renal dysfunction (Fig. 9B)

In seven studies (3RCTs, 4NRCTs), 647 patients reported the incidence of postoperative renal dysfunction^23–25,29,36,39,40^. Analyzing under the fixed effects model, the overall heterogeneity (I_2_ = 0%) is not significant. The occurrence of renal dysfunction after surgery was significantly favorable to the control (OR 2.44, 95% CI 1.12–5.28).

#### Complication: pulmonary dysfunction (Fig. 10A)

**Figure 10:**
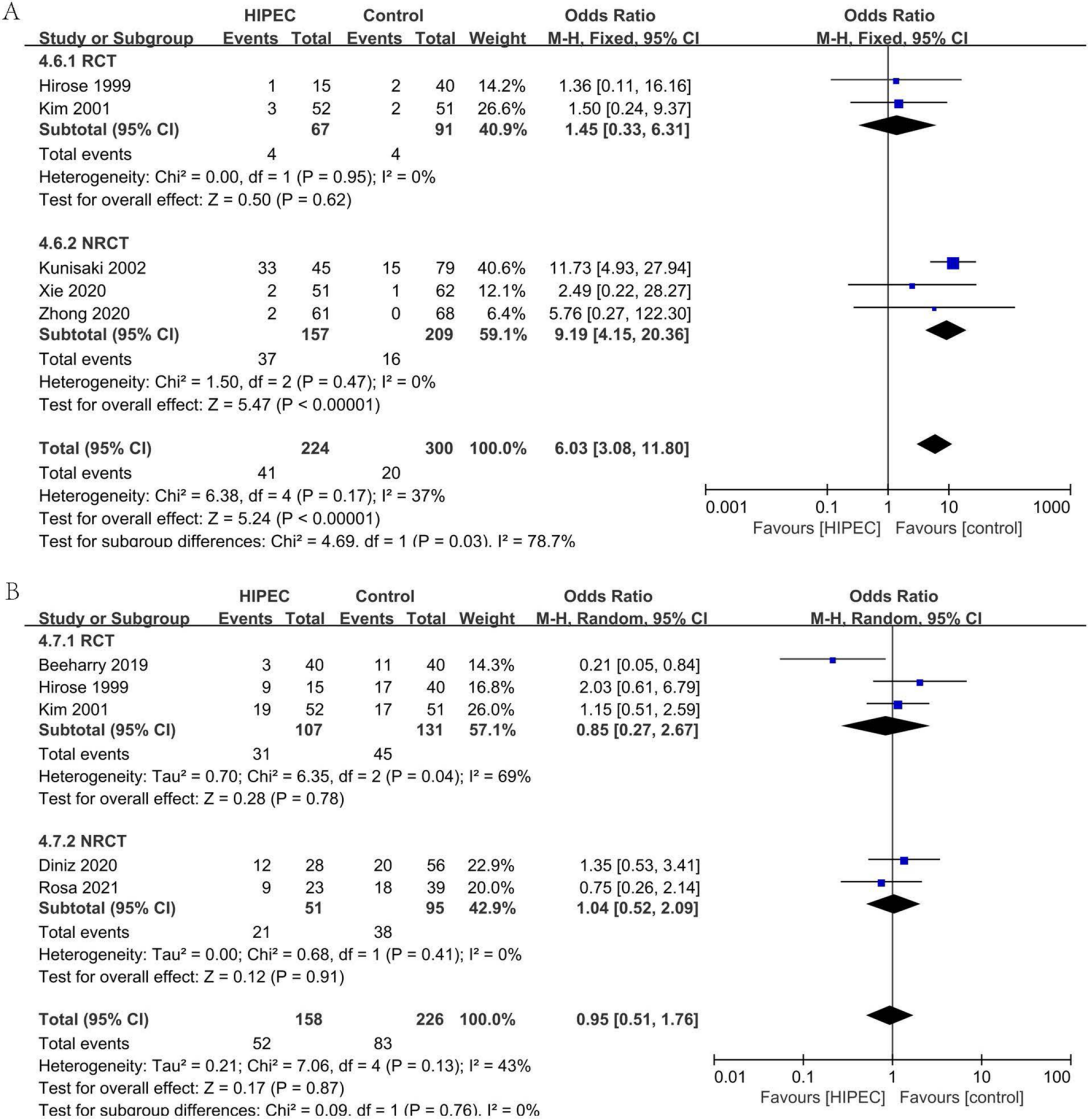
Complication: pulmonary dysfunction (**A**), Overall complication (**B**).

In five studies (2RCTs, 3NRCTs), 524 patients reported the incidence of postoperative pulmonary dysfunction^24,25,36,39,40^. Analyzing under the fixed effects model, the overall heterogeneity (I_2_ = 37%) is not significant. The occurrence of pulmonary dysfunction after surgery was significantly favorable to the control (OR 6.03, 95% CI 3.08–11.80).

#### Overall complications (Fig. 10B)

In five studies (3RCTs, 2NRCTs), 384 patients reported the incidence of overall complications^29,36,40,42,44^. Analyzing under the fixed effects model, the overall heterogeneity (I_2_ = 43%) is not significant. The overall effect is not significantly different (OR 0.95, 95% CI 0.51–1.76).

#### Deaths due to recurrence after surgery: liver recurrence (Fig. 11A)

**Figure 11:**
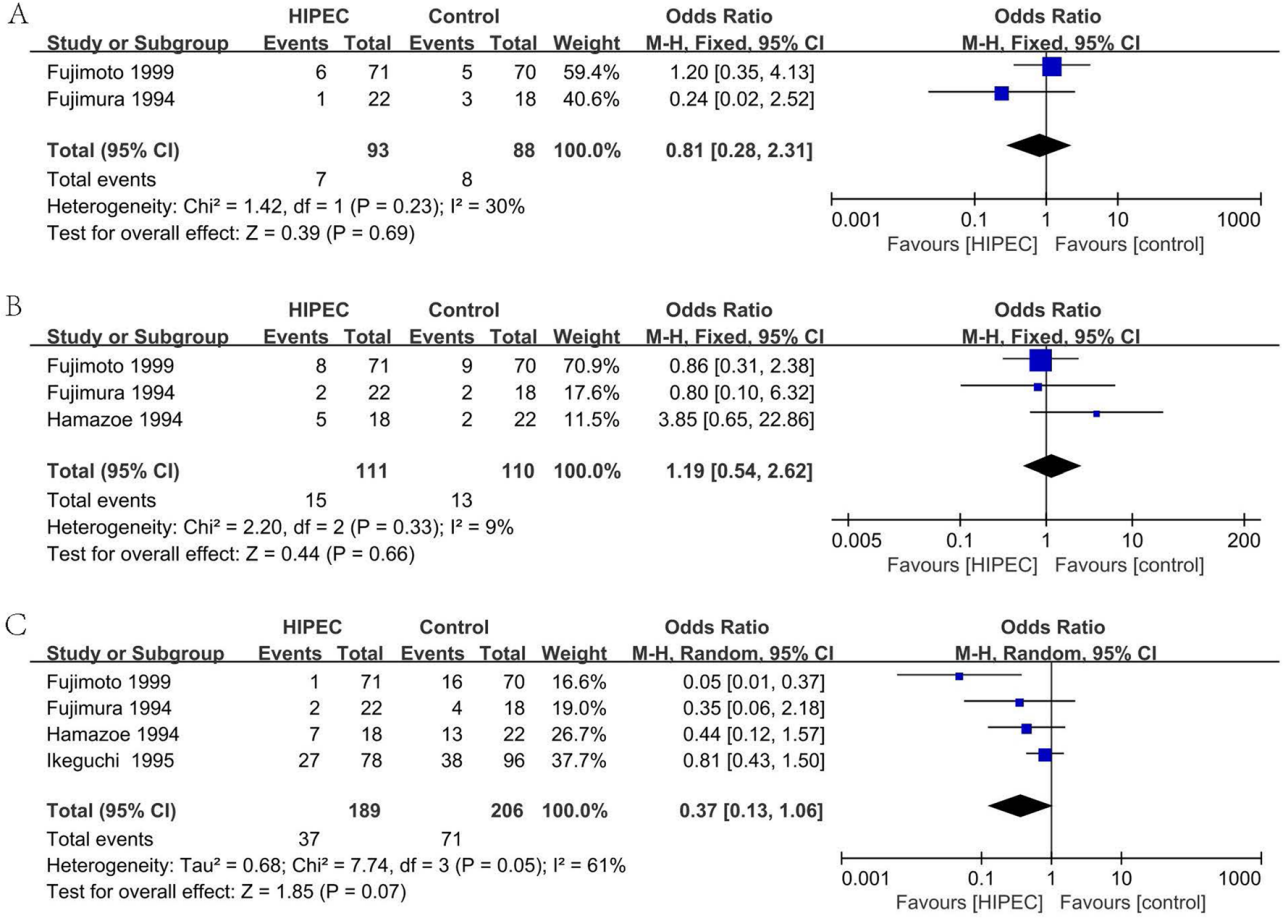
Death due to recurrence after surgery: liver recurrence (**A**), Death due to recurrence after surgery: lymph node and local recurrence (**B**), Death due to recurrence after surgery: peritoneum recurrence (**C**).

In two studies, 181 patients reported the deaths due to liver recurrence after surgery^34,35^. Analyzing under the fixed effects model, the overall heterogeneity (I_2_ = 30%) is not significant. The overall effect is not significantly different (OR 0.81, 95% CI 0.28–2.31).

#### Deaths due to recurrence after surgery: lymph node and local recurrence (Fig. 11B)

In three studies, 221 patients reported the deaths due to lymph node and local recurrence after surgery^33–35^. Analyzing under the fixed effects model, the overall heterogeneity (I_2_ = 9%) is not significant. The overall effect is not significantly different (OR 1.19, 95% CI 0.54–2.62).

#### Deaths due to recurrence after surgery: peritoneum recurrence (Fig. 11C)

In four studies, 395 patients reported the deaths due to peritoneum recurrence after surgery^33–35,37^. Analyzing under the random effects model, the overall heterogeneity (I_2_ = 61%) is not significant. The overall effect is not significantly different (OR 0.37, 95% CI 0.13–1.06).

## Discussion

For patients at high risk of peritoneal metastasis, prophylactic HIPEC after radical gastric cancer is a method to reduce peritoneal metastasis and improve the survival rate of patients, but its effect is still controversial. Our study analyzed RCTs and high-quality NRCTs to evaluate the effect of prophylactic HIPEC on long-term survival and safety of patients. This review showed that the prophylactic HIPEC is beneficial to the overall survival rate of patients at 1, 3, and 5 years, and reduces the occurrence of overall and peritoneal metastases. Our results indicate that postoperative pulmonary dysfunction and renal dysfunction are more common in the prophylactic HIPEC group. But it is regrettable that, when we evaluate deaths due to metastatic disease, HIPEC does not have enough advantages.

The overall survival rate after gastric cancer resection is a topic of concern. Many studies have reported the long-term survival rate of patients with HIPEC after surgery. Two studies reported that postoperative use of HIPEC for gastric cancer patients with peritoneal metastasis can significantly improve long-term survival^43,45^. With the increase in the incidence of gastric cancer, the effect of prophylactic HIPEC has gradually been paid attention to. In a retrospective study, Liu et al. randomly divided 128 patients into a HIPEC group and a control group. Patients in the HIPEC group received early prophylactic HIPEC + systemic chemotherapy after gastrectomy, and the control group received chemotherapy alone^26^. Through follow-up, the 1, 2, and 5-year overall survival rates of the prophylactic HIPEC group were higher than those of the control group (P < 0.05). Fujimura and his colleagues designed an RCT to evaluate the effect of prophylactic HIPEC on the overall survival rate of patients at 1, 2, and 3 years after surgery^34^. Interestingly, the author set up two experimental groups, CHPP and continuous normothermic peritoneal perfusion (CNPP), and the results reported that the overall survival rates of the two study groups were significantly different from those of the control group. A meta-analysis designed by Desidrio et al.^46^ pointed out that in the subgroup of advanced gastric cancer without peritoneal metastasis, the preventive HIPEC group had 3 years (RR 0.71, 95% CI 0.53–0.96) and 5 years (RR 0.82), 95% CI 0.70–0.96) overall survival rate is better than the control group, but there is no difference in one-year overall survival rate (RR 0.55, 95% CI 0.23–1.30). Chia et al.^47^ believe that this is because Desidrio and his colleagues did not evaluate tumor histology grades and chemotherapy regimens. Our study also reported the overall survival rate of patients at 1, 3, and 5 years after surgery. Consistent with our expected results, prophylactic HIPEC is beneficial to the survival rate of patients with gastric cancer after radical gastrectomy. And we evaluated the gastric cancer histology grade and HIPEC protocol included in this review. We conducted a subgroup analysis of the overall survival rate at 3-years of patients with different chemotherapy regimens after surgery, and the results affirm the role of prophylactic HIPEC in improving the survival rate of patients. Sun et al.’s^48^ meta-analysis included ten RCTs and concluded that HIPEC may improve the overall survival rate of patients, but it included four low-quality studies (score < 4).

There are also several studies on the choice of different chemotherapeutic drugs, but due to the small number of studies and differences in doses, the evaluation results are often limited. There is no consensus on drug selection for HIPEC, MMC and platinum drugs are more common in research. The review by Gamboa et al.^49^ summarized the choice of HIPEC chemotherapeutic drugs. According to reports, MMC is the first drug used for HIPEC monotherapy, and the most common regimen is 40 mg for 90 min. Cisplatin or oxaliplatin is usually combined with MMC. The common regimen of cisplatin is 50 to 200 mg/m^2^ 60–90 min, and oxaliplatin has a fast onset, so 460 mg/m^2^ for 30–60 min is common. In a PERISCOPE I initial results published in 2020, 460 mg/m^2^ oxaliplatin for 30 min with 50 mg/m^2^ docetaxel for 90 min is feasible^50^. But this way seems to be more suitable for patients with peritoneal metastases. Macrì et al.^51^ considered cisplatin (25 mg/m^2^ per liter) + MMC (3.3 mg/m^2^ per liter) for 60 min may be more effective. This review conducted a subgroup analysis of three different chemotherapy methods (MMC, cisplatin, MMC + cisplatin), and no matter which method they were, they improved the overall 3-year survival rate of patients. Due to the small number of studies and the differences in dose, duration, temperature, etc., we did not evaluate the effects between groups. In addition, prophylactic or therapeutic laparoscopic HIPEC has been mentioned in multiple studies. In the study of Badgwell et al.^45^, 6 patients with positive peritoneal cytology and 14 gastric cancer patients with peritoneal metastasis used laparoscopic HIPEC as preoperative adjuvant chemotherapy. At present, there is no clear standard for the selection of HIPEC chemotherapy drugs, drug dosage, duration, etc. The publication of high-quality studies can help researchers evaluate the safety and effectiveness of different chemotherapy methods^49^.

The complications of HIPEC after gastrectomy are also worrying^52^. Due to the systemic toxicity of chemotherapy drugs, patients often have complications after HIPEC. We hope that some complications are “acceptable” because they are difficult to avoid^53^. The complications of HIPEC can be divided into systemic toxicity and local toxicity. Most of the systemic toxicity is bone marrow suppression. Braam et al.^53^ pointed out that this is usually related to the dose of chemotherapy drugs. Cui et al.^28^ designed an RCT to evaluate postoperative myelosuppression. 48 patients were enrolled in the HIPEC group and the control group. Among the patients receiving HIPEC, a total of 27 patients with myelosuppression (Grade I–II: 26, Grade III–IV: 1), and 26 patients in the control group with myelosuppression (Grade I–II: 25, Grade III–IV: 1), there is no significant difference in results. In a study published in 1999, none of the 141 patients in the HIPEC group and the control group had myelosuppression. Our study included 6 literatures to evaluate the occurrence of postoperative myelosuppression, and the results were also without significantly difference. HIPEC's chemotherapy drugs are directly infused into the patient's abdominal cavity, which is different from the conventional intravenous infusion of systemic chemotherapy drugs, so the effect on the whole body may not be obvious. Anastomotic leakage and postoperative intestinal obstruction are considered to be common complications of HIPEC, and the results of this review do not seem to support this view^54–56^. Like our results, the incidence of anastomotic leakage in the HIPEC group and the control group in the meta-analysis of Desiderio et al.^46^ was not statistically significant (P = 0.63). The study by Sun et al.^48^ reported the occurrence of postoperative anastomotic leakage (P = 0.29) and intestinal obstruction (P = 0.77), and the results were also not significantly different, but the number of documents included in the analysis was small. Postoperative organ dysfunction is often reported in patients using HIPEC. In Fan et al.’s^27^ study, 36 out of 50 patients developed liver dysfunction, while Zhong et al.^24^ evaluated 129 patients and only 1 with postoperative liver dysfunction. In this meta-analysis, we evaluate liver, renal, and pulmonary dysfunction after prophylactic HIPEC. The results show that prophylactic HIPEC seems to have a limited effect on liver function, and it is more likely to cause renal dysfunction and lung dysfunction. In a meta-analysis^46^, the risk of renal dysfunction in the HIPEC group was significant (P = 0.01), which is consistent with our results. Another meta-analysis^48^ that included 10 RCTs also reported that the HIPEC group had no significant effect on liver function (P = 0.47). In the evaluation results of pulmonary dysfunction in this review, Kunisaki et al.’s^39^ research weight is relatively large (40.6%), and there is a certain degree of heterogeneity. In the study of Kunisaki et al., there are significant differences in postoperative pulmonary (73% vs 19%; P < 0.0001) and renal dysfunction (7% vs 0%; P < 0.03). The toxicity of chemotherapeutics has obvious damage to renal function and lung function. Therefore, patients with organ dysfunction should be cautious in choosing HIPEC. Although our study has no statistically significant difference in the overall risk of complications (P = 0.83), this does not mean that the risk of certain complications can be ignored, especially organ dysfunction. HIPEC is regarded as a radical therapy by many studies, therefore, whether to use HIPEC should be discussed considering the patient’s situation^19,43,57^. In order to reduce the occurrence of postoperative adverse events, the selection of patients before surgery should be decided through multidisciplinary consultation, and the appropriate treatment plan should be selected according to the principle of individualization^58^.

The metastasis of gastric cancer has a significant impact on the survival rate of patients. This review reports the effect of prophylactic HIPEC on the overall metastasis rate and peritoneal metastasis rate, confirming that prophylactic HIPEC reduces the occurrence of gastric cancer metastasis and reduces the risk of death due to peritoneal metastasis. Koemans and his colleagues pointed out in a PERISCOPE I trial that HIPEC can improve the survival rate of patients with gastric cancer, but the control of recurrence rate is not ideal^59^. This is different from our results, which may be due to different inclusion criteria and PERISCOPE I trial. Chia et al.^47^ believe that therapeutic HIPEC combined with CRS is not effective for patients with gastric cancer with peritoneal metastasis, while the effect of prophylactic HIPEC is still unclear. As an important method of perioperative chemotherapy, HIPEC is gradually recognized for its role in preventing peritoneal metastasis in advanced gastric carcinoma (AGC) patients^60^. A meta-analysis by Coccolini et al.^61^ evaluated the overall metastasis rate and peritoneal metastasis rate of patients after intraperitoneal chemotherapy (IP). A total of 8 studies were included in the overall metastasis group, and 9 studies were included in the peritoneal metastasis group. Coccolini and his colleagues reported that IP improved the overall metastasis rate of patients, and prophylactic IP significantly reduced the occurrence of peritoneal metastases. This is consistent with the results of this review. An expert consensus published in 2019 pointed out that the peritoneal metastasis of some cancers should not be regarded as end-stage disease, but localized spread^51^. This suggests that the prevention of gastric cancer peritoneal metastasis should follow the principle of local treatment under the premise of systemic treatment. At the same time, the rise of immunotherapy also provides new ideas for the treatment of gastric cancer. Catumaxomab is currently in Phase III clinical trials in China, mainly for AGC patients with peritoneal metastasis. In the future, the treatment of gastric cancer will be more individualized, so the correct evaluation of patients’ treatment methods will be an important part of tumor treatment^49^. Based on the existing evidence, we can basically affirm that preventive HIPEC can reduce the incidence of patients with peritoneal metastasis and the number of deaths due to peritoneal metastasis, but a large sample is still needed, and high-quality RCTs further evaluate the safety and the role of inhibiting disease progression of prophylactic HIPEC for patients.

This systematic review and meta-analysis contain some limitations. First, we included 10 NRCTs. Although they passed the quality assessment, this may affect the accuracy of the results. Second, China and Japan are two countries with a high incidence of gastric cancer, so there are more HIPEC-related clinical studies published^50^. We searched 3 Japanese literatures, but none of them were available. Two investigators searched the Chinese national knowledge infrastructure (CNKI) database, and we did not include them because the studies did not meet the inclusion criteria of this review or did not pass the quality assessment. In addition, there is a certain degree of heterogeneity in our research. For example, differences in patient characteristics, countries, medical levels, treatment plans, chemotherapy drugs, etc. may affect the credibility of the results.

## Conclusions

Prophylactic HIPEC may improve the survival rate of gastric cancer patients after radical gastrectomy, reduces the risk of gastric cancer metastasis, and effectively prevents peritoneal metastasis. It is recommended to select suitable patients for prophylactic use of HIPEC after multidisciplinary assessment to avoid adverse events. Large samples and high-quality clinical studies are still needed to evaluate the drug selection and dosage of prophylactic HIPEC.

## Abbreviations

PRISMA: Preferred reporting items for systematic reviews and meta-analyses
HIPEC: Hyperthermic intraperitoneal chemotherapy
GC: Gastric cancer
PC: Peritoneal cancer
MINORS: Methodological index for non-randomized studies
OR: Odds ratio
